# Editorial: The next generation of gender equality work: Reflective action for health and justice

**DOI:** 10.3389/fsoc.2022.1080349

**Published:** 2023-01-16

**Authors:** Beniamino Cislaghi, Asha L. Abeyasekera, Amiya Bhatia, Jessica K. Backman-Levy

**Affiliations:** ^1^London School of Hygiene and Tropical Medicine, London, United Kingdom; ^2^Centre for Women's Studies, University of York, York, United Kingdom; ^3^Brown School, Washington University in St. Louis, St. Louis, MO, United States

**Keywords:** gender norms, gender, equality, gender equality, systems change, power dynamics

In the last century, global advancement in programs, policies, and research on gender equality has resulted in important policy gains and expanded research agendas. Examples include: increased access for girls and women to formal education and employment, improved parental leave policies, liberation of abortion laws in a number of historically conservative countries, and increased research and programming to identify and change restrictive gender norms that lead to gender based violence, toxic masculinity, and other negative social and health outcomes. Despite this progress, key indicators of gender equality have regressed in recent years, in large part due to growing geo-political conflict, natural disasters, and the COVID-19 pandemic (World Economic Forum, 2022). In many countries, the pandemic, for example, exacerbated restrictive gender norms and triggered widening gender gaps in burden of care, paid unemployment, food security, displacement, violence (CARE, 2020), and child and early forced marriage (UNICEF, 2021), among others. In some countries, gender equality is arguably overtly under attack. The overturning of Roe vs. Wade in the United States (Howard and Krishna, 2022), the decision to further restrict abortion and make the distribution of free contraception illegal in Iran (Erfani, 2017; Mahdavi Sabet and Ghorbani, 2021), and the removal of commitments to abortion and sexual health and rights from an official UK statement on gender equality (The Guardian, 2022) are but a few of these examples. Given these current trends, we believe it important to provide rigorous evidence of the ways in which gender norms can affect the lives of all humans around the world, often (although not always) creating unequal and unjust conditions that hamper human health, wellbeing, and flourishing. Moreover, and just as importantly, we advocate for a more robust understanding of programmatic methods that center community leadership and facilitate purposeful self-reflection to reframe or loosen restrictive gender norms around the world.

The articles in this Research Topic underscore a global consensus on gender equality being a desirable moral and political goal of a contemporary just society. At the same time, they highlight how state institutions and international organizations follow the “logic of modernity” (Phillips, 2018) in achieving gender equality, namely, promoting women's empowerment *via* education, employment, and political participation, and introducing legal and policy reform to challenge harmful gender norms and address unequal power relations. The logic of modernity may appear unproblematic in its promotion of social justice, yet impose a singular linear trajectory of progress with the Global Core at the pinnacle of what the Global Peripheries must aspire toward in achieving gender equality. Transnational feminism, and more recently the emphasis on decolonizing the academy, have underscored the importance of attending to the socio-cultural differences in women's and men's experiences in various locales, paying attention to the oppression they face as well as their strengths and life chances (Marecek, 2019). The articles in this collection, while acknowledging the importance of gender equality for human flourishing, provide concerning evidence of the long-standing (and yet unaddressed) tension between universal theoretical principles of gender equality and culture-specific factors that affect the ways in which these principles are translated (or can be translated) in practice.

Cislaghi et al. draw on World Value Survey data from 97 countries to find gender inequalities in access to full time employment in most countries, and lower self-reported health among women who were not in full-time employment compared to women who were. Their findings also demonstrate that in some countries more equal gender norms did not always translate to equal access to employment and, vice versa, that women's higher full-time employment at times was registered in countries holding norms against women's participation in it. They document how the relationship between women's full-time employment rates and pro-equality gender norms is deeply influenced by country-specific historical, cultural, and socio-economic factors. Another two empirical explorations are Mejía-Guevara et al.'s study of women's use of family planning in India and Coll et al.'s investigation of mother's whose daughters experienced FGM/C in several countries in Africa. Mejía-Guevara et al. draw on nationally representative data in India to provide evidence that men's attitudinal norms at the community level about contraceptive use and beliefs that they control their wife decrease women's contraceptive use, and these associations persist even when accounting for women's empowerment at the individual and district level. Coll et al. first demonstrate links between women's empowerment and being against the continution of FGM/C, and then show how girls with mothers who have high empowerment and do not support FGM/C are less likely to experience FGM/C. All three studies underscore how gender norms can shape health outcomes and offer methodological approaches to using existing data to generate evidence on links between gender norms, gender equality, and health. The studies also further demonstrate how the “logic of modernity” is not self-evident. Women exercise agency by reinterpreting existing gender norms; this can be in the form of resistance (O'hanlon, 1988) or even by reinhabiting norms (Mahmood, 2005) and are shaped by their aspirations, desires, and life-projects (which do not necessarily align with the aspirations and desires that global health policy makers hold for them).

The articles in this collection also provide evidence of change in systems of norms, suggesting that if change is to be sustainable, it needs to be achieved through full community engagement. Studying the case of water management in rural Tanzania, Eaton et al. show that a critical mass for change emerges when people occupying varied community roles join in transformative critical reflections on the existing gender order and the potential benefits of transforming it. Their study also underscores the importance of working with both men and women: positive models of masculinity helped women participate in their husbands' water management tasks. Similarly, Bergenfeld et al. suggest that tribes, family, and peers have a critical role to play in changing norms related to reporting sexual harassment against women in Jordan, demonstrating the importance of engaging all members of a community when addressing gender based violence.

In addition to demonstrating the importance of working with entire communities (rather than people individually) to change gender norms, this special issue includes two articles inviting reflexivity in gender equality practice. Lokot's paper draws attention to the work cultures of international, pro-equality organizations that include gender norms, practices, and relations that can contradict the principles they promote through their programmes. Similarly, McCarthy et al. study the case of the construction industry in the United Kingdom (historically dominated by white men) to show how employees' perception of their organization's commitment to justice in general was crucial in reducing resistance to company gender equality policies.

Numerous cases exist of both spontaneous and intervention-facilitated transformations of gender norms that benefit people of all genders. The articles in this special collection are but a few of these examples. As work toward gender equality expands, however, there is an urgent need to critically examine the biases and colonial practices absorbed from the patriarchal and capitalist hegemonic order that structure research and action (Cislaghi et al., forthcoming; Grey, 2004; Ladner, 2009; Dietze, 2014; Nkenkana, 2015). Global and local program planners, policy makers, and researchers must engage in our own critical self-reflection and examine the role that institutions in the industrialized Global “Core” (rather than the geographical Global North) have played in setting the discursive agenda at the expense of those in the Global “Periphery.” As anti-equality movements strengthen, we must design the next generation of programs, policies, and research on gender norms and gender equality to challenge the power hierarchies and resource environments that drive the exclusion of minoritized scholars, activists, and practitioners. In doing so, we create opportunities to change and refocus the questions we are asking and center the voices of those who may feel bound and restricted by the very norms this work seeks to change.

## Author contributions

All authors collaborated on the conception and writing of this editorial and read, revised, and approved the submitted version.
